# The efficacy of conventional and technology assisted cue exposure therapy for treating substance use disorders: a qualitative systematic review

**DOI:** 10.3389/fpsyt.2025.1544763

**Published:** 2025-03-26

**Authors:** Daniel Thaysen-Petersen, Sigurd Krogh Hammerum, Signe Wegmann Düring, Pia Veldt Larsen, Anders Fink-Jensen, Angelina I. Mellentin

**Affiliations:** ^1^ Psychiatric Center Copenhagen, Frederiksberg Hospital, Mental Health Services, Capitol Region Hospitals, Frederiksberg, Denmark; ^2^ Psychiatric Center Sct. Hans, Mental Health Services, Capitol Region Hospitals, Roskilde, Denmark; ^3^ Department of Clinical Medicine, Faculty of Health and Medical Sciences, University of Copenhagen, Copenhagen, Denmark; ^4^ Psychiatric Center Amager, Mental Health Services, Capitol Region Hospitals, Copenhagen, Denmark; ^5^ Mental Health Services, University of Southern Denmark, Vejle, Denmark; ^6^ Unit for Clinical Alcohol Research (UCAR), Unit for Psychiatric Research, Department of Clinical Research, University of Southern Denmark, Odense, Denmark; ^7^ Department of Psychiatry, Odense University Hospital, Region of Southern Denmark, Odense, Denmark; ^8^ Brain Research-Inter-Disciplinary Guided Excellence (BRIDGE), Department of Clinical Research, University of Southern Denmark, Odense, Denmark; ^9^ Center for Digital Psychiatry (CDP), Mental Health Services in the Region of Southern Denmark, Odense, Denmark

**Keywords:** cue exposure therapy, substance use disorders (SUDs), systematic review, technology-assisted, virtual reality

## Abstract

**Background:**

Cue Exposure Therapy (CET) is a behavioristic psychological intervention for treating substance use disorders (SUDs). Recently, CET has been examined in technology-assisted formats to increase intervention efficacy. No systematic review has examined the efficacy of different CET formats across types of SUDs.

**Objectives:**

We aimed to examine the efficacy of CET across SUDs and examine the efficacy of non-technology-assisted (NT-CET) and technology-assisted CET (T-CET).

**Methods:**

We conducted a systematic literature search in MEDLINE, PsycINFO, EMBASE, and the Cochrane Central Register of Controlled Trials up to June 2024. The efficacy of CET was inspected trough a qualitative synthesis and the quality assessment of all the included studies was performed using the Cochrane risk-of-bias tool for randomized trials, version 2.

**Results:**

Forty-four controlled trials were identified (NT-CET; n=21; T-CET: n=23). Most studies were conducted on alcohol- and nicotine use disorders. No study reported effect sizes on craving, while one study reported a small effect of NT-CET on alcohol consumption at 6- and 12-months follow-up. Compared to control interventions, CET was found more effective in 41% of the studies that examined cravings, and in 57% of the studies that examined consumption. In these studies, there was on overrepresentation of studies that combined CET with cognitive behavioral therapy (CBT) or CBT-related approaches. Only one study directly compared the effect of NT- and T-CET alcohol craving or consumption and found no difference up to 6 months follow-up. Among NT-CET and T-CET studies, the proportion of studies reporting significantly better outcomes than control interventions were 17% and 60% for craving, respectively, and 38% and 80% for consumption, respectively. High heterogeneity and risk of bias were found among the included studies.

**Conclusions:**

Across the different substance use disorders, most studies found significant reduction in craving and consumption after CET. No conclusions can be made on the efficacy of CET compared to active control interventions, due to limiting reporting of effect sizes. Technology-assisted CET reported significant reduction in craving and consumption relatively more often than conventional CET studies, particularly when delivered in virtual reality. Future high-quality studies are warranted to enable more firm conclusions and quantitative synthesis.

**Systematic review registration:**

https://www.crd.york.ac.uk, identifier CRD42022308806.

## 1 Introduction

Substance use disorders (SUDs) are complex brain disorders that either directly or indirectly cause more than 11.8 million deaths annually worldwide (1, 2). Psychological interventions are key components in the treatment strategies for SUD. These interventions are in some cases combined with pharmacological treatment, but even when combined, the efficacy remains limited (3).

According to dual process models of addiction, addictive behavior is influenced by the interaction between an impulsive and a reflective cognitive system (4). The impulsive system is based on an automatic evaluation of immediate short-term outcomes in terms of rewards vs. consequences. The reflective system is based on controlled evaluation of long-term outcomes from recall of memories, knowledge, and higher cognitive processes allowing self-regulation (4–6). Dual process models propose that individuals with SUDs have an imbalance between these two cognitive systems. The impulsive system is over-activated and sensitized towards the drug of choice and associated cues. Meanwhile, the reflective system is relatively under-activated and thus, unable to regulate responses from the impulsive system (7). Since the impulsive system is partly automatic and implicit, responses to cues in the environment may lead to the maintenance of substance abuse despite explicit knowledge about the consequences generated by the reflective system (4, 5, 8).

Conventional evidence-based psychological interventions for SUDs such as motivational interviewing (MI) and cognitive behavioral therapy (CBT) rely mainly on the modification of reflective cognitive processes, while impulsive cognitive dysfunctions are addressed to a much lesser extent (9–11). Cue exposure therapy (CET) is a behavioristic psychological approach based on classical conditioning that targets the overactive impulsive system (12–14). During CET, patients with SUD are exposed to substances or substance-associated cues *in vivo* to elicit cravings while refraining from habitual behavioral responses, i.e., consumption. With repeated exposure, the sensitization of the impulsive system is assumed to decrease (13, 14).

CET has been combined with other techniques, including imaginary CET (thinking about substances and substance-related cues), urge-specific coping skills (USCS) and CBT (12, 15). However, although CET has been combined with a variety of techniques, the intervention has generally shown limited efficacy concerning reducing cravings and consumption across different types of SUDs (12, 15, 16). The limited efficacy of *in vivo* CET may be due to its inability to provoke sufficient cue-induced cravings when delivered in clinical settings (12). Also, conventional CET usually exposes patients to simple cues, e.g., a bottle of preferred beverage, and not substance-associated situations (complex cues). Therefore, conventional CET may not sufficiently eliminate cravings activated by the complex cues that individuals with SUDs encounter outside clinical settings, e.g., at home, bars, restaurants, supermarkets, and other drug-related environments (12, 17). Using technology to assist the delivery of CET (technology-assisted CET (T-CET)) has been proposed as a solution to increase efficacy. T-CET encompasses all digital formats that assist *in vivo* exposure to substances and substance-associated cues including computers, projectors, apps, and VR. Several studies have shown that T-CET can effectively provoke cue-induced cravings and offer exposure to complex cues (16–19), however, no previous study has compared conventional non-technology-assisted CET (NT-CET) and T-CET.

To our knowledge, no previous systematic review has examined the efficacy of different CET delivery formats across different types of SUDs. Therefore, the aims of this qualitative systematic review were 1) to examine the efficacy of CET in reducing cravings and consumption across SUD types; and 2) to examine the efficacy of conventional non-technology-assisted CET (NT-CET) and T-CET in reducing cravings and consumption.

## 2 Methods

### 2.1 Protocol registration and reporting

The protocol was registered in the International Prospective Register of Systemic Reviews (PROSPERO): registration no. CRD42022308806; available at https://www.crd.york.ac.uk/prospero/display_record.php?RecordID=308806. The reporting of this systematic review was guided by The Preferred Reporting Items for Systematic Reviews and Meta-Analyses (PRISMA) Statement (20).

### 2.2 Eligibility criteria

To be eligible for inclusion in the present systematic review, studies had to 1) be original studies written in English and published in peer-reviewed journals; 2) be at least controlled trials (CT) and preferably randomized controlled trials (RCTs) that examined the effects of CET *in vivo* on changes in craving level and/or consumption of the substance; 3) have been conducted on adults aged ≥ 18 years from sub-clinical or clinical populations who had a diagnosis of SUD according to any version of the International Classification of Diseases (ICD) or Diagnostic and Statistical Manual of Mental Disorders (DSM); a population was also considered to be clinical if it was examined in a treatment setting and/or described as comprising “patients” according to diagnostic nomenclature. Studies were excluded if the participants had severe psychiatric- or neurological comorbidity (e.g., psychotic- or bipolar disorders, intellectual disability, dementia, or brain damage).

### 2.3 Electronic databases and literature search

A systematic literature search was conducted in MEDLINE (via PubMed), PsycINFO (via APA), EMBASE (via Ovid), and the Cochrane Central Register of Controlled Trials (CENTRAL), with no limitations regarding the year of publication, up to June 2024. The search was performed using a PICO (Population, Intervention, Control, Outcome) search strategy composed of three major blocks: 1) substance use disorder (population); 2) cue exposure therapy (intervention); and 3) consumption and cravings (outcomes). Detailed search strategies are presented in **Appendix 1**.

### 2.4 Study selection

The study selection was conducted using the review management software Covidence. Two authors (DT-P and SKH) independently screened the titles and/or abstracts of the studies identified by the electronic database searches and excluded those that did not meet the eligibility criteria. Subsequently, the same two authors independently performed a *full-text screening* of the remaining studies. In cases of disagreement about the eligibility of studies, the full-text screening was performed by a third researcher (AIM). The reference lists of the selected articles were also screened to identify any other relevant studies not identified by the electronic database searches.

### 2.5 Data extraction

Two authors (DT-P and SKH) independently extracted data from the included studies. In cases of disagreement or need for supervision, a third author assisted with the data extraction (AIM). The following study characteristics were extracted from the individual studies, if available: author and publication year, study design, average age, gender, SUD population (sub-clinical or clinical), treatment setting, diagnostic SUD assessment, experimental and control interventions, number of participants allocated to experimental and control groups, treatment as usual (psychological and/or pharmacological treatment), treatment goal (abstinence, moderation, reduction of cravings), outcome measures (cravings and consumption), measurement time points, and main findings.

### 2.6 Qualitative synthesis

The characteristics of the included studies are presented in **Supplementary Data Sheet 2**. The efficacy of CET was inspected trough a qualitative synthesis by categorizing the studies into the following groups: 1) significantly superior to control interventions, 2) significantly inferior to controls interventions, or 3) non-significant. Stacked bar charts were produced to illustrate the proportions of studies in each category. Additional stacked bar charts were produced to account for differences in sample size, where the proportions of total number of patients in each category was illustrated. The bar charts were further divided into each SUD subtype (alcohol use disorder (AUD), nicotine use disorder (NUD) or other SUD’s) as well as delivery format (NT-CET, T-CET including VR, and only VR). Finally, a sensitivity analysis including studies with sample size ≥ 15 patients in each group (control and intervention groups) was conducted (**Appendix 2**).

A meta-analysis was not conducted due to the high heterogeneity among the 44 included studies. The studies varied considerably within the CET interventions, co-interventions, control interventions, number of treatment sessions, pharmacological treatments, and treatment settings. Given these substantial differences, pooling effect sizes quantitatively would not have yielded a meaningful or reliable synthesis of the findings.

### 2.7 Risk of bias

The quality assessment of the included studies was performed using the Cochrane risk-of-bias tool for randomized trials, version 2 (ROB-2). The ROB-2 provides a framework for evaluating individual items pertaining to the following five domains: 1) Selection bias: due to the randomization process; 2) Performance bias: due to deviations from the intended interventions; 3) Attrition bias: due to missing outcome data; 4) Detection bias: due to measurement of the outcome; and 5) Reporting bias: due to selection of the reported results (65). For all the included studies, each domain was given a rating of “low risk of bias”, “some concerns”, or “high risk of bias”, and an overall risk of bias score was generated based on the scores for the five domains. Two authors (DT-P and SKH) independently scored the items pertaining to each domain, scored each domain, and generated an overall risk of bias score under the supervision of a third author (AIM).

## 3 Results

### 3.1 Study selection

**Figure 1:**
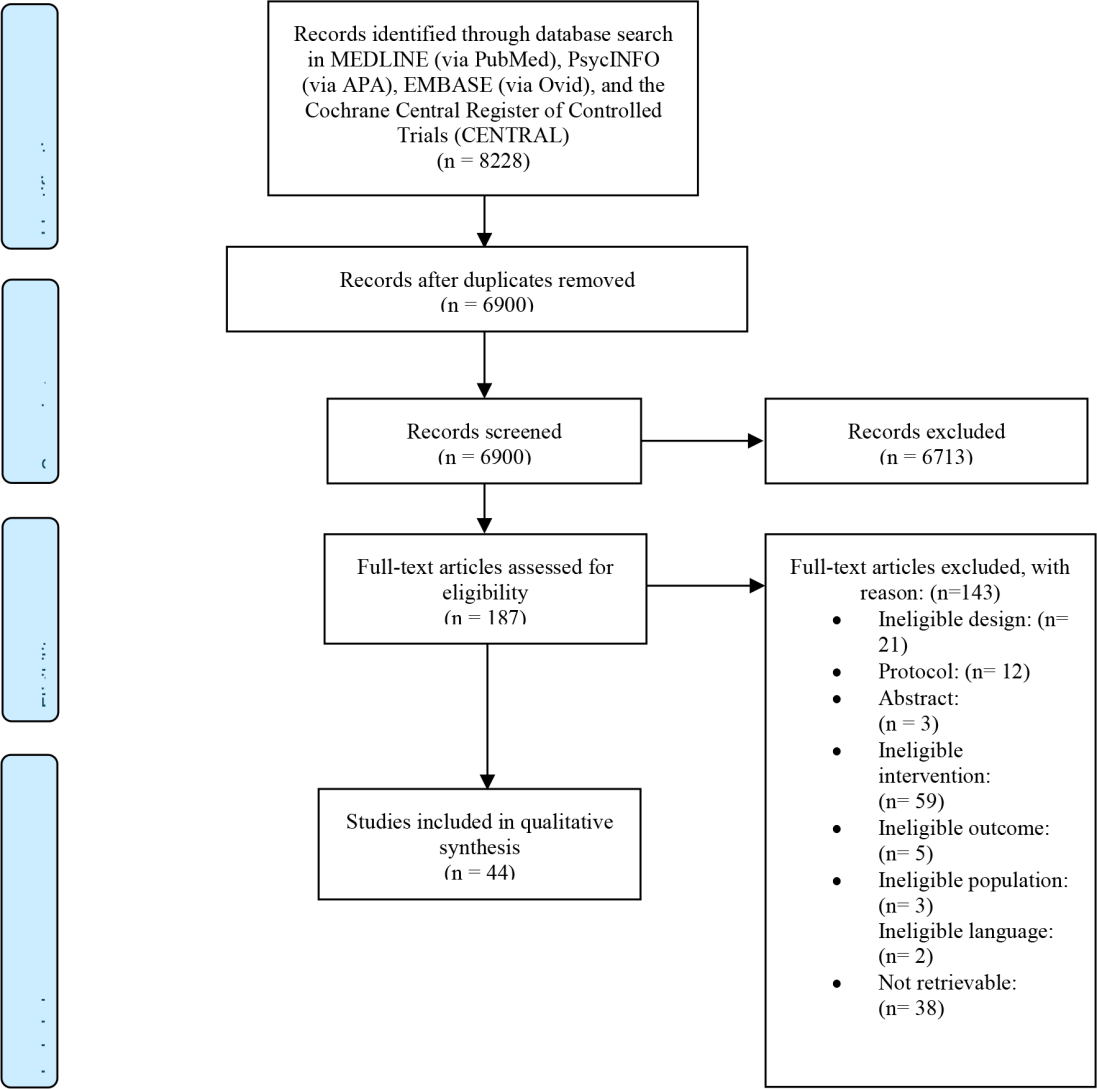
Flow chart illustrating study selection process.

The database searches yielded a total of 8228 records. Manual searches of the references cited in published original studies and review articles did not yield any additional studies. After removal of duplicates, a total of 6900 studies remained. The titles and/or abstracts of these studies were screened leading to the exclusion of 6713 studies. The remaining 187 studies were assessed for eligibility based on full-text reading. After full text reading, 143 studies were excluded, leaving a total of 44 studies that were selected for inclusion. The study selection process is illustrated in **Figure 1**.

### 3.2 Study characteristics

**Supplementary Data Sheet 2** presents a summary of the characteristics of the included studies, the measures used, and the main findings. The included studies were either RCTs (n=35) or non-randomized controlled trials (CTs) (n=9) (**Supplementary Data Sheet 2**). Twenty-two studies were conducted on samples with AUD (21–33, 42–50, 58), 14 on samples with NUD (34–38, 51–59), four on samples with opioid use disorder (OUD) (39, 40, 63, 64), and three on samples with cocaine or other stimulant use disorders (CSUD): cocaine (n=1) (60), methamphetamine (n=2) (61, 62). One study was conducted on a sample with a mix of SUDs (41).

A total of 21 studies applied NT-CET: AUD samples (n=13) (21–33), NUD samples (n=5) (34–38), OUD samples (n=2) (39, 40) and a mix of SUDs sample (41). The remaining 23 studies applied T-CET: AUD samples (n=9) (42–44, 49, 50), NUD samples (n=9) (51–59), CSUD samples (n=3) (60–62), and OUD samples (n=2) (63, 64). Across the included studies, the average sample size was 65 participants (range: 10 to 165). Most of the studies included both male and female participants (range: 0-100% males), while 11 studies only included males, and one study only included females. Overall, males were generally overrepresented in the included samples. The average age of the participants was 39.8 years (range: 23 to 54.6 years). The majority of the studies recruited participants via advertisement or from inpatient or outpatient settings.

#### 3.2.1 SUD assessment

Regarding the studies conducted on clinical SUD samples, SUDs were assessed using the Structured Clinical Interview for DSM (SCID) (66–68)) in four studies (32, 52, 55, 64), and the Composite International Diagnostic Interview (CIDI) (69) in one study (28). Different versions of the interviews were used depending on the diagnostic nomenclature applied in the studies, including the International Statistical Classification of Mental and Behavioral Disorders, Tenth Revision (ICD-10) (World Health Organization (70) as well as the Diagnostic and Statistical Manual of Mental Disorders, 3^rd^, 4^th^, and 5^th^ editions (DSM-III, DSM-IV and DSM-5) (71). A total of 33 clinical studies assessed SUDs using clinical diagnostic assessment, where six studies relied on the ICD-10 (42, 43, 45, 47, 50, 62), four studies on the DSM-III (24, 26, 30, 31), 11 studies on the DSM-IV (28, 29, 32, 33, 40, 41, 49, 52, 53, 55, 64), and six studies on the DSM-5 (44, 46, 48, 54, 58, 61). The remaining eight clinical SUD samples did not report the assessment method, but since the included patients were either in treatment for or described as having a SUD, we deem it appropriate to categorize the patients as having SUD diagnoses.

Nine studies were conducted on sub-clinical SUD samples, all including NUD studies. Regarding the sub-clinical NUD samples, the assessment was performed using the Fägerström Test for Nicotine Dependence (FTND) (72) in two studies (51, 59) and minimum number of cigarettes/day in two studies (56, 57). Five studies did not report the assessment method (34–38). These sub-clinical populations were included, since the patients were described as having symptoms of dependence. Sixteen of the clinical studies were conducted in inpatient settings, 14 were conducted in outpatient settings, four in both inpatient and outpatient settings, and one did not report the treatment setting (**Supplementary Data Sheet 2**). Of the sub-clinical populations, four studies did not report the treatment setting, while the remaining studies were conducted in outpatient clinics (n=4), or in a laboratory setting (n=1).

#### 3.2.2 Experimental and control groups

Across the experimental and control groups, the average number of sessions was 8.6 (range: 4 to 32 sessions), with a mean session duration of 55.7 mins (range: 20 to 120 mins). The average length of treatment was 5.3 weeks (range: 0.7 to 16 weeks).

##### 3.2.2.1 Experimental group

In six studies, the experimental group only received *in vivo* CET. To intensify the intervention, the remaining 38 studied combined *in vivo* CET with multiple different co-interventions: imaginal CET was the most frequent add-on (n=14), followed by USCS (n=8), priming and response prevention (PRP) (n=5), CBT (n=3), aversion (n=2), MI (n=1), crushing of cigarettes (n=1), mobile application group chat support (n=1), text message feedback or reminders (n=2), phone call (n=1), biofeedback therapy (n=1), communication skills training (CST) (n=1), and deep muscle relaxation (n=1).

###### 3.2.2.1.1 NT-CET

Twenty-one studies applied NT-CET, and all of these studies exposed patients to substances in real life. Most NT-CET studies applied simple cues (n=19/21), while only two of the NT-CET studies applied complex cues either by conducting the exposure in a simulated bar (AUD sample) (26) or by accompanying patients to places rich in drug-associated memories and cues (OUD sample) (39) (**Supplementary Data Sheet 2**).

Within the 21 NT-CET studies, the 13 AUD samples applied NT-CET. Among these, most interventions consisted of patients looking, holding and/or smelling the alcoholic beverage (n=12/13), while one study did not specify the exposure process. In five of the AUD studies, the subjects also drank a small amount of the alcoholic beverage as a part of the exposure i.e., priming (21, 23, 25, 27, 73). Among the five NUD samples applying NT-CET, the patients were instructed to either look and hold (n=2) (34, 35), look (n=1) (37), look, smell and smoke a sham cigarette (n=1) (36), or look, hold, smell, and take a puff without inhaling (n=1) (38). Among the two OUD samples applying NT-CET, one study instructed patients to look at photographs of drug use as well as look and hold paraphernalia (39), while one study instructed patients to look, hold, and smell heroin (40). The study including patients with different SUD subtypes instructed patients to look at drug related cues (41).

###### 3.2.2.1.2 T-CET

Twenty-three studies applied T-CET. Most of these studies applied VR (n=14), and the remaining nine studies used computers, video, monitors/projectors, or an app on a mobile phone. Most T-CET studies applied complex cues (n=20/23), while only three of the T-CET studies applied simple cues either by exposing patients to alcoholic beverages on a monitor or app on a mobile phone (42, 43), or by instructing patients to find and crush virtual cigarettes in a medieval castle in VR (51).

The 20 T-CET studies applying complex cues included a variety of different approaches: AUD studies (n=7): three studies exposed patients to complex cues on a computer or projector (46, 48, 50); four studies exposed patients to alcohol-associated environments in VR including a restaurant (Italian and Chinese), bar, pub, beer garden, and whiskey house all including a variety of alcoholic beverages (beer, soju, whiskey and wine) (44, 45, 47, 49). While two of the VR studies instructed patients to either say no to alcohol (47) or choose a non-alcoholic beverage as fast as possible (46), one study exposed patients to someone vomiting after the cue exposure (49). NUD studies (n=8): one study exposed patients to up to nine personal smoking-related images on a computer (57); seven studies exposed patients to smoking-related environments in VR (52–56, 58, 59) including a party, driving a car, inside and outside of a restaurant, office building and courtyard, convenience store, airport smoking lounge and gate, modern apartment with outdoor seating, replica of Venice beach, waiting at a bus stop in Los Angeles, coffee shop, pub, at home (lunch, breakfast, watching TV), waiting in the street, and a beach bar. Other SUDs (n=5): One study exposed patients with cocaine use disorder to a videotape of a person administering cocaine (60); two studies exposed patients with methamphetamine use disorder to either a women administering methamphetamine in VR (62) or methamphetamine-related situations in VR followed by environments with individuals were being arrested, experienced hallucinations, had infections and skin ulcers, contracted sexually transmitted diseases, lost a tooth or died suddenly (61); two studies exposed patients with opioid use disorder to videos with either a man smoking or injecting heroin (64), or individuals seeking and buying opioids, inhaling opioids, experiencing withdrawal and overdose (63).

##### 3.2.2.2 Control group

In 20 studies, the control group received treatment-as-usual (TAU) only, as described in the next section. In 12 studies, TAU was combined with add-on treatments: sham CET applying *in vivo* neutral stimuli (n=5), meditation or relaxation techniques (n=4), CBT (n=1), imaginal CET (n=1), or aftercare as usual (n=1). In the remaining studies (n=12), the control groups received CBT alone (n=6), imaginal CET (n=1), rapid smoking alone (n=1) or combined with MI and USCS (n=1), a self-help cessation manual (n=1), placebo psychotherapy (n=1), or no treatment (n=1).

#### 3.2.3 Treatment as usual

In 66% of the included studies (n=29/44), the experimental groups received CET as an add-on to treatment as usual (TAU). The TAU primarily consisted of psychological therapy (n=24), while five studies only applied pharmacological treatment as TAU, and five studies applied psychological and pharmacological treatments as TAU. The psychological treatment consisted of MI, CBT, or behavioral therapy (BT) (e.g., contingency management or role play to practice drink refusal skills). Other psychological, social, and environmental treatments (OPSET) were also used (e.g., social skills training, education on harmful use, social reintegration, psychodrama, family-, couples-, occupational- and vocational counseling, supportive-expressive psychotherapy, community meetings, self-help manuals, lifestyle changes, legal education, exercise) as well as 12-step programs (12SP). The most frequently applied psychological treatment was CBT alone (n=11), sometimes/occasionally combined with MI (n=1) or BT (n=1). OPSET, consisting mainly of non-evidence-based interventions, was applied in many studies (n=17) and was in some studies combined with 12SP (n=4) or CBT+BT (n=1). Pharmacological treatment consisted of nicotine replacement therapy (NRT), benzodiazepines, disulfiram, and anti-craving agents, such as naltrexone and acamprosate, applied alone or in combination with psychological treatment. The remaining studies did not report whether the sample received pharmacotherapy as TAU (n=12).

#### 3.2.4 Treatment goal

Across all the studies, the treatment goal was either a reduction in craving (n=17), consumption (n=12), or a combination of craving and consumption (n=15). Regarding the studies that examined the effect of CET on consumption (n=27), the treatment goal was reduced consumption/moderation in 13 studies and abstinence in 14 studies.

### 3.3 Outcome measures and follow-up

#### 3.3.1 Craving measures

To assess changes in cravings, the studies used a variety of questionnaires, with some of them being SUD specific. Among the studies conducted on AUD samples, the following alcohol-specific instruments were used: Alcohol Urge Questionnaire [AUQ: (73)], Alcohol Craving Questionnaire [ACQ: (74), and Multidimensional Alcohol Craving Scale – Virtual reality (MACS-VR: (75)]. The studies conducted on NUD samples used the following instruments: Questionnaire of Smoking Urges [QSU: (76)], Urge To Smoke Scale [UTS: (77)]; Tobacco Craving Questionnaire [TCQ: (78)], and French Tobacco Craving Questionnaire [FTCQ-12: (58)]. Regarding other SUD diagnoses often involving illegal drugs (COUD and OUD), more generic instruments were used: Desires for Drug Questionnaire [DDQ: (79)]. Ten studies used visual analogue scales [VAS: (80)], including VAS-101, VAS-11, and VAS-100. In the remaining craving questionnaires, the items were developed by the research-groups and were typically assessed using Likert scales or numeric rating scales.

#### 3.3.2 Consumption measures

Across SUDs, relatively few studies applied psychometrically sound consumption measures, such as the daily estimation calendar methods: Timeline Follow-back method [TLFB): (81)] and Form 90 (82), or quantity-frequency methods: Problem Drinking Questionnaire (83). The remaining studies applied data from patient journals, patient diaries, standardized assessment developed by the research team (SART), and some studies even evaluated consumption without any standardization or at least no description of the measure. Very few studies attempted to validate self-reported consumption using collateral informants or biomarkers.

There was variability in the operationalization from self-reported questionnaires. The most common outcomes were abstinence, drinking/drug use days, drinks/amount per drinking/drug using day, heavy drinking days, and time to relapse to any drinking/drug use.

#### 3.3.3 Timepoints for follow-up

The time points for outcome assessment varied. Most studies performed assessments at the final session (n=40), while later follow-up (FU) assessments were conducted at one month FU (n=8), two months FU (n=5), three months FU (n=16), six months FU (n=18), and 12 months FU (n=6). Less frequent FU timepoints were applied at six weeks FU (n=1), four months FU (n=3), five months FU (n=2), eight months FU (n=1), and nine months FU (n=1).

### 3.4 Main results

#### 3.4.1 Craving

Approximately 70% (n=32) of the included studies assessed cravings, with 30 studies comparing within-group changes and 27 studies comparing changes between groups. None of the included studies reported the effect size of CET on craving to control interventions.

Across all SUDs, the within-group analyses revealed a reduction in cravings in 1) both the experimental and control groups in 17 studies (57%), 2) only in the experimental group in nine studies (30%), and 3) only in the control group in one study (3%). Three studies (10%) found reduced cravings in both groups. None of the included studies reported an increase in cravings.

**Figure 2:**
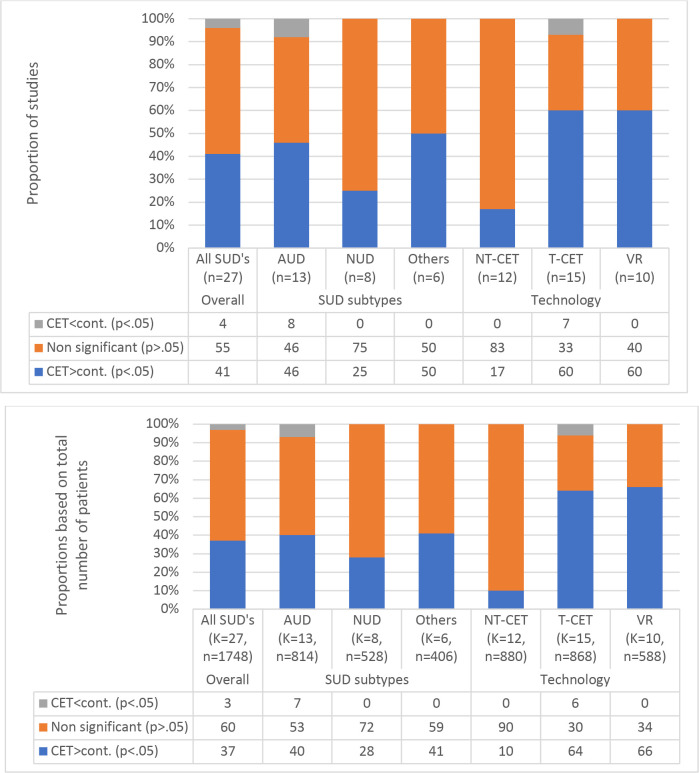
The proportions of statistically significant findings and the direction of the findings when comparing cue exposure therapy (CET) and control interventions on craving outcomes across substance use disorders and delivery formats.

A graphical illustration of the between-group comparisons is presented in **Figure 2**. Across all SUDs, 11 studies found a greater reduction of CET (41%), one study (4%) found a greater reduction in cravings in the control group, and 15 studies (55%) found no statistically significant difference in cravings between CET control interventions. Regarding the different types of SUDs, the results of the between-group analyses varied considerably. The CET group achieved a greater reduction in cravings compared to the control group in 46% of the AUD studies, 50% of the studies conducted on samples with other SUDs (heroin and methamphetamine), and 25% of the NUD studies. Findings were similar when comparisons were based on the total number of patients (**Figure 2**).

In the 11 studies that demonstrated a superior effect of CET compared to control interventions regarding reduction in cravings, significant reductions were found at the final treatment session in 10 studies and at three months FU in one study. The average number of CET sessions in these 11 studies was 7.7 (range: 5-12), with a mean session duration of 50.5 mins (range: 8-90 mins), and the sessions were conducted within an average of 4.5 weeks (range: 1-10 weeks). The experimental interventions consisted of *in vivo* CET alone (n=4) or in combination with aversion (n=2), CBT (n=1), USCS (n=1), MI (n=1), imaginal CET + biofeedback therapy (n=1) or phone call or text message reminder (n=1). The control interventions consisted of either TAU alone (n=9), TAU + sham CET with neutral cues (n=1), or CBT alone (n=1). Regarding the risk of bias, all studies either had high risk (n=6) or some concern (n=5).

In the single study that demonstrated an inferior effect of CET compared to the control intervention regarding reduced cravings, patients in both groups received six 50-minute sessions two weeks. The experimental intervention involved showing the patients pictures of their preferred alcoholic beverages while the patients in the control group were shown pictures of a cleaning agent (42). The study was assessed as having a low risk of bias.

In the 15 studies that found no statistically significant difference between the CET and control interventions regarding reduction in cravings, the average number of CET sessions was 9.7 (range: 4-16), with a mean session duration of 51 mins (range: 25-90 mins), and the sessions were conducted within an average of 5.5 weeks (range: 2-10 weeks). The studies applied CET alone (n=6) or in combination with either imaginal CET (n=3), imaginal CET + USCS (n=2), imaginal CET + PRP + recall unpleasant experiences (n=1), imaginal CET + CBT + nicotine gum (n=1), or USCS (n=1). In these studies, the control interventions consisted of TAU alone (n=4), sham CET with neutral cues (n=2), meditation and relaxation training (n=2), CBT (n=3), CBT + nicotine gum (n=1), imaginal CET (n=1), rapid smoking + MI + USCS (n=1), or OPSET (n=1). Regarding bias risk, studies either reported high risk (n=8) or some concern (n=7).

As illustrated in **Figure 2**, the between-group analyses revealed a noticeable difference when comparing NT-CET (n=12) and T-CET (n=15) studies. A higher proportion of the T-CET studies (60%) than the NT-CET studies (17%) found a greater reduction in cravings in the experimental group compared to the control groups. Most NT-CET studies included in the between-group analysis (n=10/12) applied simple cues, whereas most T-CET studies applied complex cues (n=14/15). When T-CET studies were further sub-categorized into VR studies only, all applied complex cues. However, there was no additional effect on the proportion of studies showing a greater reduction in cravings after CET compared to the control groups (60%)

The sensitivity analyses, which only include studies with ≥ 15 patients in both the intervention and control groups, confirm findings presented in **Figure 2** (see **Appendix 2**).

#### 3.4.2 Consumption

Sixty-eight percent (n=28) of the included studies assessed consumption, with 15 studies comparing within-group changes and 23 studies comparing changes between groups. Only one study reported the effect size of CET on consumption and found a small effect (32).

Across all SUDs, the within-group analyses showed a reduction in consumption in both the experimental and control groups in 12 studies (80%). In comparison, two studies only found a reduction in the experimental group (13%), and one reported increased consumption in both groups (7%).

**Figure 3:**
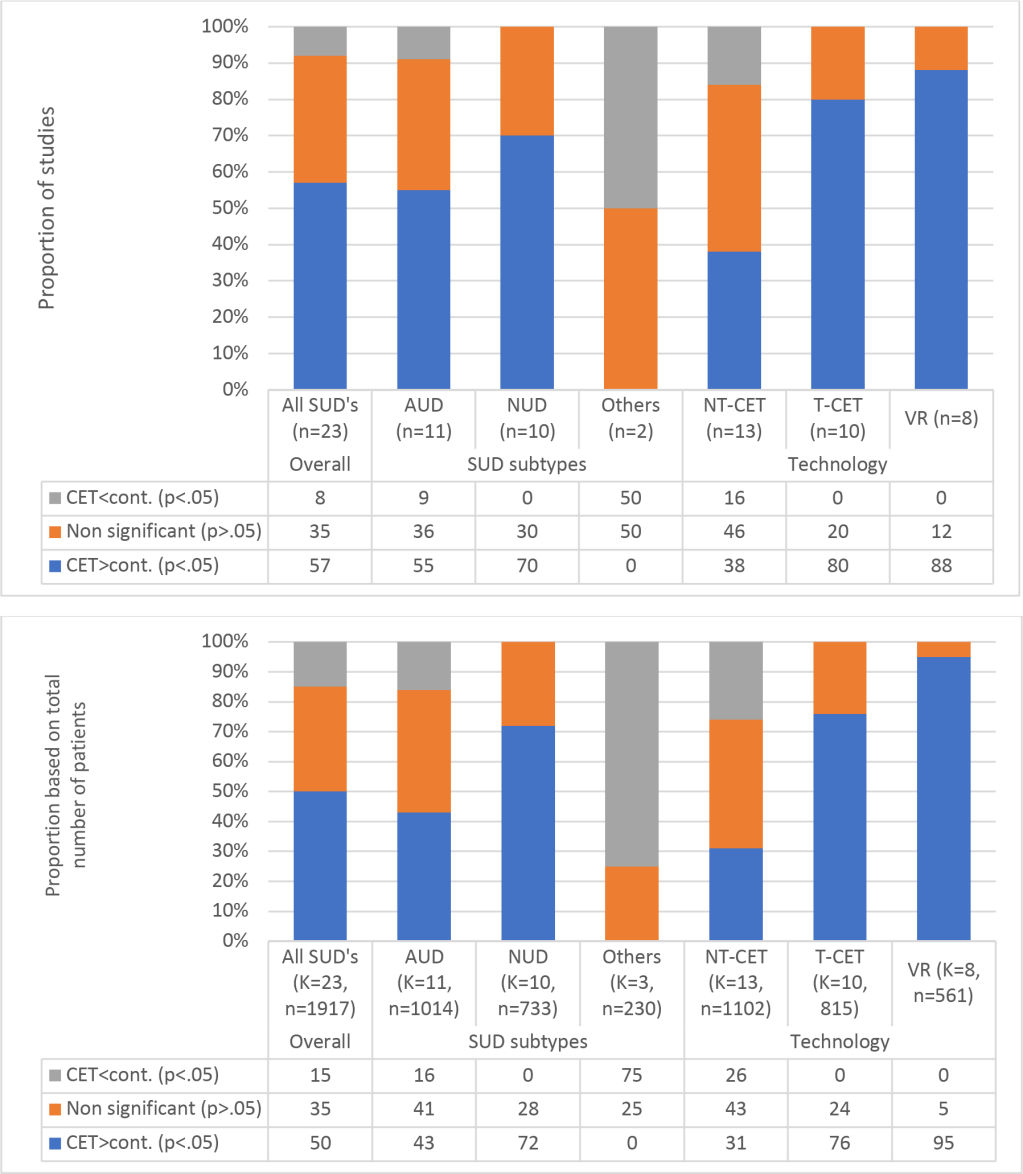
The proportions of statistically significant findings and the direction of the findings when comparing cue exposure therapy (CET) and control interventions on consumption outcomes across substance use disorders and delivery.

A graphical illustration of the between-group comparisons is presented in **Figure 3**. Across all SUDs, 13 studies (57%) found CET to be superior to control interventions in reducing consumption. In comparison, two studies (8%) demonstrated an inferior effect of CET compared to control interventions, and eight studies (35%) found no statistically significant difference in consumption between experimental and control groups. Regarding different types of SUDs, the findings varied considerably. The experimental group achieved a greater reduction in consumption compared to the control group in 70% of the NUD studies, 55% of AUD studies, and none of the studies conducted on samples with other SUDs (opioids). Findings were similar when comparisons were based on the total number of patients (**Figure 3**).

In the 13 studies that demonstrated a superior effect of CET compared to control interventions regarding reduction in consumption, CET demonstrated significant reductions at post-treatment (NUD: n=5), two months FU (NUD: n=1), three months FU (NUD: n=1; AUD: n=1), four months FU (NUD: n=1), six months FU (AUD: n=5; NUD: n=2), and 12 months FU (AUD: n=1; NUD: n=1). The average number of CET sessions in these 13 studies was 9.5 (range: 4-21), with a mean session duration of 53.8 mins (range: 20-90 mins), and the sessions were conducted within an average of 6.1 weeks (range: 10 days - 12 weeks). The experimental interventions consisted of CET alone (n=4) or in combination with imaginal CET + USCS (n=1), imaginal CET + USCS + CST (n=1), CBT (n=1), USCS (n=1), USCS + rapid smoking (n=1), mindfulness + peer-to-peer support (n=1), PRP (n=1), phone call or text message reminder (n=1), or crushing cigarettes (n=1). The control interventions consisted of either TAU only (n=7), sham CET with neutral cues (n=2), CBT (n=1), meditation and relaxation (n=1), relaxation (n=1), or self-help manual (n=1). Regarding the risk of bias, all studies had some concern (n=7) or high risk (n=6).

The two studies that demonstrated an inferior effect of CET compared to control interventions regarding consumption reduction combined CET with either imaginal CET + PRP + recall unpleasant experiences (8 sessions, 17 min/session, conducted within 10 weeks) or USCS (9 sessions, 60 mins/session, conducted within 3 weeks). The control interventions either TAU (CBT) or OPSET. The risk of bias was some concerns (n=1) and high (n=1).

In the eight studies that found no statistically significant difference between the CET and control interventions regarding consumption reduction, the average number of CET sessions was 9.1 (range: 4-16), with a mean session duration of 64.6 mins (range: 25-90 mins), and the sessions were conducted within an average of 6.6 weeks (range: 3-16 weeks). The experimental intervention consisted of CET alone (n=2) or including either PRP (n=2), imaginal CET + USCS (n=2), imaginal CET + CBT + nicotine gum (n=1), or imaginal CET (n=1). In these studies, the control interventions consisted of CBT (n=4), CBT + nicotine gum (n=1), TAU alone (n=1), rapid smoking + MI + USCS (n=1), or aftercare as usual (n=1). Regarding the risk of bias, all studies were either high risk (n=4), some concern (n=3), or low (n=1).

When comparing NT-CET (n=13) and T-CET studies (n=10), the between-group analyses for changes in consumption showed a similar pattern as for cravings but even more favorable for T-CET. A higher proportion of the T-CET studies (80%) than the NT-CET studies (38%) found a greater reduction in consumption in the experimental group compared to the control group. As described previously, most of the NT-CET studies (n=11/13) applied simple cues, whereas most T-CET studies applied complex cues (n=8/10). When selecting the VR studies, all applied complex cues. An even higher proportion of these studies found a greater reduction in consumption in the experimental group compared to the control group (88%).

The sensitivity analyses, which only include studies with ≥ 15 patients in both the intervention and control groups, confirm the findings presented in **Figure 3** (see **Appendix 2**).

#### 3.4.3 Studies with craving and consumption as outcomes

A total of 16 studies assessed both cravings and consumption. However, to inspect whether studies with a statistically significant reduction in cravings after exposure to CET also observed a statistically significant decrease in consumption, only studies that performed within-group analyses were of interest. We identified eight studies that examined within-group changes in both cravings and consumption. Four studies were conducted on NUD samples and four studies on AUD samples. Almost every study (n=7) found a significant within-group reduction in both cravings and consumption. Of these, four studies applied NT-CET, while three applied T-CET in VR. The one study that reported a decrease in consumption but not craving applied NT-CET and was conducted on a NUD sample (38).

### 3.5 Risk of bias in individual studies

The results of the risk of bias assessment are summarized in **Table 1**. Regarding the overall risk of bias, 25 of the 44 included studies were classified as having a “high risk of bias”, 17 studies were classified as having “some concerns”, and only two studies were classified as having a “low risk of bias”. Selection-, attrition-, and reporting bias were predominantly classified as “low risk of bias” (28/44, 25/44, 40/44, respectively), performance bias as “some concerns” (23/44) or “high risk of bias” (17/44), and detection bias as “low risk of bias” (22/44) or “some concerns” (17/44).

**Table 1:** Risk of bias.

Author/year	Selection	Performance	Attrition	Detection	Reporting	Overall
Alcohol use disorder (n=22)						
Rankin et al. (1983) (21)	Some concern	Some concern	Low	High	Low	High
McCusker et al. (1995) (22)	Low	Some concern	Low	Low	Low	Some concern
Sitharthan et al. (1997) (23)	Some concern	Some concern	Low	Low	Low	Some concern
Staiger et al. (1999) (24)	Low	Low	Some concern	Low	Low	Some concern
Heather et al. (2000) (25)	Low	Some concern	Some concern	Low	Low	Some concern
Rohsenow et al. (2001) (26)	Low	Some concern	Some concern	Low	Low	Some concern
Dawe et al. (2002) (27)	Some concern	Some concern	Some concern	Low	Low	Some concern
Kavanagh et al. (2006) (28)	Low	Some concern	Some concern	Some concern	Low	Some concern
Vollstädt-Klein et al. (2011) (29)	Low	Some concern	Low	Low	Low	Some concern
Monti et al. (1993) (30)	Low	Some concern	Some concern	Some concern	Low	Some concern
Drummond et al. (1994) (31)	High	Some concern	Low	Some concern	Low	High
Monti et al. (2001) (32)	High	Some concern	Low	Low	Low	High
Loeber et al. (2006) (33)	High	High	Low	Low	Low	High
Geisel et al. (2016) (42)	Low	Low	Low	Low	Low	Low
Mellentin et al. (2019) (43)	Low	Low	Low	Low	Low	Low
Hernandes-Serrano et al. (2020) (44)	Low	Low	Some concern	High	Low	High
Zhang et al. (2023) (45)	Low	High	High	Low	Some concern	High
Weber et al. (2023) (46)	High	High	Some concern	High	High	High
Thaysen-Petersen et al. (2023) (47)	High	Some concern	Low	Low	Low	High
Ng et al. (2024) (48)	Low	Some concern	Low	High	Low	High
Lee et al. (2009) (49)	High	High	Low	Low	Low	High
Nattala et al. (2018) (50)	High	Some concern	Low	Low	Low	High
Nicotine use disorder (n=13)						
Corty et al. (1984) (34)	Some concern	High	Low	Some concern	Low	High
Brandon et al. (1987) (35)	Low	Some concern	Some concern	Low	Low	Some concern
Niaura (1999) (36)	Low	Some concern	Some concern	Some concern	Low	Some concern
K. P. Morganstern (1969) (37)	High	High	Some concern	High	High	High
Raw et al. (1979) (38)	High	High	Low	Some concern	Low	High
Girard et al. (2009) (51)	Low	High	High	Some concern	Low	High
Bordnick et al. (2012) (52)	Low	Some concern	Low	Some concern	Low	Some concern
Culbertson et al. (2012) (53)	Some concern	Some concern	Low	Some concern	Low	Some concern
Park et al. (2014) (59)	High	Some concern	Low	Some concern	Low	High
Malbos et al. (2018) (54)	Low	High	High	Some concern	Low	High
Pericot-Valverde et al. (2019) (55)	Low	Some concern	Low	Some concern	Low	Some concern
Goldenhersch et al. (2020) (56)	Low	Some concern	Some concern	Some concern	Low	Some concern
Pollak et al. (2021) (57)	Low	High	Low	Some concern	Low	High
Malbos et al. (2022) (58)	Low	High	High	Some concern	Low	High
Opioid use disorder (n=4)						
Dawe et al. (1993) (39)	Low	High	High	Some concern	Low	High
Marissen et al. (2007) (40)	Low	High	Low	Some concern	Low	High
de Quirós Aragón et al. (2005) (63)	Low	High	High	Low	Low	High
Du et al. (2014) (64)	Low	High	Low	Low	Low	High
Mix of substance use disorders (n=1)						
Havermans et al. (2007) (41)	Low	Some concern	High	Low	Low	Some concerns
Cocaine and other stimulant use disorders (n=3)						
O’Brien et al. (1990) (60)	Some concern	High	Low	Low	Some	High
Wang et al. (2019) (61)	Low	High	Low	Low	Low	High
Ji et al. (2023) (62)	Low	Some concern	Low	Low	Low	Some concern

## 4 Discussion

### 4.1 Principal findings

This is the first systematic review to examine the efficacy of different CET delivery formats on craving and consumption outcomes across various types of SUDs. Across all SUDs, CET was found to be more effective than active control interventions in reducing cravings in 46% of the studies and consumption in 57% of the studies. There were considerable differences when the data was analyzed based on CET-delivery format: NT-CET or T-CET. In the NT-CET studies, a greater reduction was found for the experimental group compared to the control group in 23% and 38% of the studies for craving and consumption, respectively. T-CET studies reported a greater reduction in the experimental group compared to the control group in 67% and 80% of the studies for craving and consumption, respectively. Unfortunately, there were several limitations of the included literature: The overall risk of bias was high, and the included studies were heterogeneous in terms of CET regimens, add-on treatments, control interventions, TAU, number of sessions, interval between session, assessment methods, and follow-up periods.

Previous literature reviews that examined CET have exclusively investigated changes in consumption (12, 15, 16, 84). These reviews found no consistent evidence for the efficacy of CET for treating SUDs overall (12, 16), or found that CET showed no to medium effects when targeting AUD (15, 84). Nonetheless, it should be noted that the studies included in the prior reviews on AUD samples compared CET to active control interventions that often consisted of the most effective evidence-based treatments, such as CBT or CBT-related approaches (USCS, third wave CBT approaches as mindfulness, etc.) (15, 84). In addition, several systematic reviews have examined the efficacy of T-CET, but they only included VR studies targeting either AUD alone (18, 85) or patients with SUD types (86, 87). These prior reviews included more heterogeneous samples and study designs compared to the present review, such as including participants aged ≤ 18 years, samples with behavioral addictions (e.g., internet gaming disorder), and non-controlled trials. Furthermore, additional studies examining T-CET targeting SUDs have been published since these reviews were conducted (45–48, 62). The present review sought to systematically address a broader research question concerning the impact of delivery formats by including controlled trials that examined the efficacy of both NT-CET and T-CET (including VR).

The present study implemented more conservative eligibility criteria compared to prior reviews: we only included studies conducted on adults aged ≥ 18 years of age, the sub-clinical and clinical samples only fulfilled the diagnostic criteria for a SUD and not any other diagnosis, and we focused explicitly on self-reported cravings and consumption as outcomes. Although one of the main aims of the present review was to examine the efficacy of CET across several types of SUDs, most of the existing studies, particularly the T-CET studies, only examined AUD and NUD samples. In addition, the included studies only assessed changes in craving in the short term, and thus, the long-term efficacy of CET on cravings remains to be investigated. Consumption was assessed at a variety of time points up to 12 months FU, although the majority only included short-term FU up to 6 months.

The present review shows that CET reduced cravings and consumption in most of the included studies. Furthermore, the effect of CET was either superior to or non-significantly different from active control interventions in almost all studies. In the studies reporting the best effect of CET compared to active controls on craving measures, CET was often combined with MI or CBT-related interventions (CBT and USCS) and compared non-evidence-based interventions. The control interventions non-significantly different from CET usually included MI, CBT or CBT-related interventions (USCS, mindfulness, etc.). These qualitative findings support the quantitative results of Mellentin et al. and Kiyak et al., suggesting that CET alone is inferior to CET combined with USCS or CBT alone (15, 84). Considering dual process models, the advantage of combining CET with CBT-related treatments might be that CET targets the impulsive system, whereas CBT-related interventions target the reflective system. Thus, this combination of treatments targets both cognitive systems, which might restore the balance between them, enabling the reflective system to regulate impulsive responses and prevent relapse. Seven studies directly compared CET and CBT (23, 25, 27, 33, 36, 49, 59). While five of these studies found no difference between the interventions, two studies showed better efficacy from CET on craving (49) or consumption (23). None of the included studies found a better effect from CBT compared to CET.

Regarding bias, most studies had an overall moderate to high risk of bias. Within the subcategories, selection, attrition, detection, and reporting bias were mainly assessed as low risk of bias or with some concern. However, in terms of performance bias, a substantial part of the studies was assessed as having some concern or high risk due to difficulties of blinding participants to interventions. No pattern was found in the relation between efficacy from CET and bias assessments. Furthermore, the significant heterogeneity regarding populations, CET regimens, and control interventions adds to the risk of confounding bias, which may compromise to the qualitative findings.

Across the different types of SUDs, the effect of CET on craving and consumption measures varied. While most AUD samples experienced a significant effect on craving, most NUD samples experienced a significant effect on consumption. Even though these proportional differences cannot be translated directly into differences in terms of efficacy, the findings are contrary to previous speculations that NUD samples are less responsive to CET due to cigarettes being smoked in a broader range of contexts than other substances and therefore have an increased risk of reinstatement (12). A possible explanation may be that most NUD studies used complex cues in VR, which translate more effectively to real-world contexts, i.e., higher ecological validity. Very few studies have been conducted on patients using substances other than alcohol and nicotine. Therefore, the qualitative results found in the present study is primarily based on CET regimens in these populations. One study that examined patients with OUD (heroin dependence) should be mentioned due to its paradoxical findings (40). In this specific study, patients allocated to CET experienced a much greater relapse rate compared to those assigned to psychotherapy.

Although CET is based on principles of extinction learning that are broadly applicable across substance use disorders, it is important to recognize that different substances may elicit distinct neurobiological and behavioral responses to treatment. As highlighted by Karoly et al. (2015), alcohol, nicotine, and opioids share common neural pathways related to addiction, yet they also exhibit unique pharmacological effects and reinforcement mechanisms (88). This heterogeneity may influence the efficacy of CET, potentially impacting craving reduction and relapse prevention differently depending on the substance in question. To address these differences, we categorized studies by SUD subtype (alcohol use disorder, nicotine use disorder, and other SUDs) in our synthesis and visualizations. However, the variability in CET efficacy across substances remains an important consideration. Future research should focus on substance-specific differences in CET response, potentially adapting exposure protocols to align with the unique characteristics of different addictions. Understanding these nuances will be critical for optimizing CET as a targeted intervention for specific SUD populations.

Previous reviews have suggested that technological advances may improve the efficacy of CET. The present review found results that support this proposition. When compared to control interventions, the proportion of studies reporting a significant reduction in craving and consumption was more than twice as high for T-CET than NT-CET studies. Furthermore, when CET was delivered using VR, a slightly greater proportion of studies reported a favorable effect of CET on consumption. A possible reason for this difference maybe that T-CET provides exposure to complex cues with greater ecological validity.

Interestingly, only one directly compared T-CET and NT-CET. Unfortunately, in the study comparing NT-CET and T-CET, simple cues were applied in both groups (T-CET: exposure to pictures of alcohol beverages on smartphone vs. NT-CET: exposure to usually consumed beverage in real-life), and no difference was found up to 6 months FU (43). Two studies applied complex cues during NT-CET to investigate the effect of applying complex during conventional NT-CET in real life (28, 39). The complex cues were delivered by creating a room that resembled a bar (28) or by accompanying patients to locations rich in drug-associated memories or populated by drug users (39). However, the results did not favor CET: One of the studies found a significantly better effect on consumption in the control group compared to CET (28), while none of the studies found any difference in craving between CET and controls. In these two studies, both the CET and control groups were also treated with CBT as TAU, which might have saturated the treatment efficacy. It is noteworthy that in one of the studies, the exposure to complex cues could not be completed for six of 18 opioid-dependent patients due to safety concerns (39). Thus, even when exposing patients to complex cues in an environment with great ecological validity, the efficacy might not be superior to other evidence-based interventions. The findings also highlight that the delivery of complex cues in conventional CET exhibits safety concerns. None of the included studies directly compared exposure to simple and complex cues.

The integration of T-CET into clinical practice presents both opportunities and challenges that must be addressed for effective implementation. Key factors include accessibility, cost, clinician training, ethical considerations, and patient engagement. A major consideration is technological infrastructure: VR-based exposure therapy requires specialized hardware and software, which may not be feasible in resource-limited settings. Mobile and computer-based alternatives are more accessible but still require stable internet, adequate screen resolution, and secure data storage. Cost is another barrier: VR equipment and software entail expenses for maintenance, updates, and licensing. Clinicians and institutions must weigh benefits against costs and consider reimbursement policies. Financial support from healthcare systems or research grants may be needed for long-term sustainability. Effective implementation also requires clinician training: Providers must be proficient in both traditional cue exposure therapy and the use of technology-based tools. Training should focus managing patient reactions and integrating technology into treatment plans to avoid underutilization or misapplication. Ethical and privacy concerns must also be addressed: Digital platforms collecting patient data must comply with regulations such as HIPAA (in the U.S.) or GDPR (in Europe). T-CET may also trigger distress, requiring clear protocols for monitoring and providing support. Patient engagement is another critical aspect: While some individuals find technology-enhanced therapy appealing, others may struggle with new technology or prefer traditional methods. Strategies such as real-time clinician support, gamification, and personalized exposure scenarios may enhance adherence. Finally, T-CET must be integrated into existing treatment protocols: This includes combining it with evidence-based treatment, e.g., CBT, MI, and medication-assisted treatment, and ensuring it complements rather than replaces in-person therapy. Standardized guidelines are still emerging, and further research is needed to develop best practices. Thus, T-CET shows great promise but requires careful planning to address infrastructure, cost, training, ethics, engagement, and clinical integration.

### 4.2 Strengths and limitations

The present systematic review has several strengths, including pre-registration of the study protocol, extensive electronic database searches, adherence to PRISMA guidelines, and inclusion of controlled trials only. Also, a number of limitations pertaining to the individual trials weaken the conclusions of this study: 1) heterogeneity regarding populations, follow-up time points, CET regimens, control interventions, and, in particular, outcome assessments and endpoints which might have confounded the results; 2) limited studies include participants with other SUD, 3) no objective assessments of cravings or consumption; 4) risk of bias, particularly performance bias, was observed in 40 out of 44 studies, where there was had either “some concern” or “high risk of bias” due to unblinded participants

Since outcomes are highly heterogeneously reported, we deemed it appropriate to synthesize the results qualitatively; therefore, no firm conclusions can be made regarding efficacy Thus, results from the present study must be considered in the context of the methodological approach, the considerable heterogeneity of the included studies, and the high level of bias.

### 4.3 Conclusions and future directions

In conclusion, most studies found a significant reduction in craving and consumption after CET across SUDs. When CET was compared to control interventions, most studies found CET to be either superior or comparable in terms of reducing craving and consumption. T-CET, particularly when delivered in virtual reality, was superior to control interventions in a higher proportion of studies, compared to NT-CET. This difference may be a result of NT-CET studies predominantly applying simple cues, while most T-CET studies applied complex cues. Among the studies reporting significantly better outcomes from CET on craving and consumption, the majority of studies compared CET to non-evidence-based control interventions. On the contrary, the studies that found no difference in the effect on craving and consumption often compared CET to evidence-based control interventions, e.g., CBT. Based on the considerable methodological heterogeneity and high degree of bias, these results must be interpreted with caution. In order to make more firm conclusion on the efficacy of CET, and the relative efficacy from NT-CET and T-CET, more high-quality research is needed. In particular, studies including more homogeneous populations and interventions, as well as directly comparing NT-CET and T-CET are warranted.

## Data Availability

The original contributions presented in the study are included in the article/**Supplementary Material**. Further inquiries can be directed to the corresponding author.

## Glossary

ACQ: Alcohol Craving Questionnaire
AUD: Alcohol use disorder
AUQ: Alcohol urge questionnaire
BT: Behavioral therapies
CBT: Cognitive behavioral therapy
CET: Cue exposure therapy
CIDI: Composite international diagnostic interview
CST: Communication skills training
CSUD: Cocaine or other stimulant use disorders
CT: Controlled trial
DDQ: Desires for Drug Questionnaire
DSM: Diagnostic and statistical manual of mental disorders
FTCQ: French Tobacco Craving Questionnaire
FTND: Fägerström test for nicotine dependence
FU: Follow-up
ICD: International classification of diseases
MACS-VR: Multidimensional alcohol craving scale –
MI: Motivational interviewing
NRT: Nicotine replacement therapy
NT-CET: Non-technology cue exposure therapy
NUD: Nicotine use disorder
OPSET: Other psychological, social, and environmental treatments
OUD: Opioid use disorder
PRISMA: Preferred reporting items for systematic reviews and meta-analyses
PRP: Priming and response prevention
QSU: Questionnaire of smoking urges
RCT: Randomized controlled trials
ROB-2: Risk of bias - version 2
SADQ: Severity of alcohol dependence questionnaire
SART: Standardized assessment developed by the research team
SCID: Structured clinical interview for DSM
SUD: Substance use disorder
TAU: Treatment as usual
T-CET: Technology-assisted cue exposure therapy
TCQ: Tobacco craving questionnaire
TLFB: Timeline follow back method
USCS: Urge-specific coping-skills
UTS: Urge to smoke scale
VAS: Visual analogue scales
VR: Virtual reality
12SP: 12-step programs.
