# Supplementary material for: The efficacy of conventional and technology assisted cue exposure therapy for treating substance use disorders: a qualitative systematic review
Source: Front Psychiatry. 2025 Mar 26;16:1544763. doi: 10.3389/fpsyt.2025.1544763 (PMC11979113; doi:10.3389/fpsyt.2025.1544763)
Supplement: Supplementary file 1 [file DataSheet1.docx]

**Appendix 1** Search strategy

PubMed:

("Alcohol use disorder*"[tw] OR "AUD"[tw] OR "Alcohol Related Disorder*"[tw] OR "Alcohol-Related Disorder*"[tw] OR "Alcohol Induced Disorder*"[tw] OR "Alcohol-Induced Disorder*"[tw] OR "Alcohol-Dependen*"[tw] OR "Ethanol Dependen*"[tw] OR "Alcohol Abuse"[tw] OR "Ethanol Abuse" [tw] OR "Alcohol Addict*"[tw] OR "Alcohol Dependen*"[tw] OR "Alcohol Misuse"[tw] OR "Harmful Use of Alcohol"[tw] OR "Chronic Alcoholi*"[tw] OR "Alcoholic Intoxication"[tw] OR "Alcohol Intoxication"[tw] OR "Alcohol-Intoxication"[tw] OR "Binge Drink*"[tw] OR "Alcoholi*"[tw] OR "Problem Drinker"[tw] OR "Problematic Drinker"[tw] OR "Alcohol-Related Disorders"[Mesh] OR "Alcoholics"[Mesh] OR "SUD"[tw] OR "Drug abuse"[tw] OR "Drug addiction"[tw] OR "Cocaine"[tw] OR "Heroin"[tw] OR "Opioid*"[tw] OR "Nicotine"[tw] OR "cannabis"[tw] OR "tobacco"[tw] OR "TUD"[tw] OR "Addictive disorders"[tw] OR "addiction"[tw] OR "substance use disorders"[tw] OR "Substance Addiction"[tw] OR "Substance Dependence"[tw] OR "Drug Use"[tw] OR "Substance-Related Disorders"[tw] OR "Substance-Related Disorders"[Mesh]) AND ("Cue Exposure Therap*"[tw] OR "Cue exposure intervention*"[tw] OR "CET"[tw] OR "Exposure therap*"[tw] OR "Exposure intervention*"[tw] OR "Conditioning Therap*"[tw] OR "Conditioning intervention*"[tw] OR "Cue Exposure" [tw] OR "VR"[tw] OR "Virtual reality"[tw] OR "Desensitization, Psychologic"[tw] OR "Implosive Therapy"[tw] OR "Virtual Reality Exposure Therap*"[tw] OR "Virtual Reality Therap*"[tw] OR "Desensitization, Psychologic"[Mesh])) AND ("Craving"[tw] OR "Drive"[tw] OR "Motivation"[tw] OR "Alcohol drinking"[tw] OR "Alcohol consumption"[tw] OR "Alcohol intake"[tw] OR "Cocaine consumption"[tw] OR "Cocaine intake"[tw] OR "Cannabis consumption"[tw] OR "Cannabis intake"[tw] OR "Opioid consumption"[tw] OR "Opioid intake"[tw] OR "Amphetamine consumption"[tw] OR "Amphetamine intake"[tw] OR "Nicotine consumption"[tw] OR "Nicotine intake"[tw] OR "Ketamine consumption"[tw] OR "Ketamine intake" [tw] OR "Benzodiazepine consumption"[tw] OR "Benzodiazepine intake"[tw] OR "Benzodiazepine intake"[tw] OR "Drinking behavior"[tw] OR "Smoking"[tw] OR "Marijuana use"[tw] OR "drug-seeking behavior"[tw] OR "Motivation" [Mesh] OR "Drinking behavior"[Mesh] OR "Smoking"[Mesh] OR"Marijuana use"[Mesh] OR "drug-seeking behavior"[Mesh])

Psycinfo:

("Alcohol use disorder*" OR "AUD" OR "Alcohol Related Disorder*" OR "Alcohol-Related Disorder*" OR "Alcohol Induced Disorder*" OR "Alcohol-Induced Disorder*" OR "Alcohol-Dependen*" OR "Ethanol Dependen*" OR "Alcohol Abuse" OR "Ethanol Abuse" OR "Alcohol Addict*" OR "Alcohol Dependen*" OR "Alcohol Misuse" OR "Harmful Use of Alcohol" OR "Chronic Alcoholi*" OR "Alcoholic Intoxication" OR "Alcohol Intoxication" OR "Alcohol-Intoxication" OR "Binge Drink*" OR "Alcoholi*" OR "Problem Drinker" OR "Problematic Drinker" OR "Alcohol-Related Disorders" OR "Alcoholics" OR "SUD" OR "Drug abuse" OR "Drug addiction" OR "Cocaine" OR "Heroin" OR "Opioid*" OR "Nicotine" OR "cannabis" OR "tobacco" OR "TUD" OR "Addictive disorders" OR "addiction" OR "substance use disorders" OR "Substance Addiction" OR "Substance Dependence" OR "Drug Use" OR "Substance-Related Disorders" OR "Substance-Related Disorders") AND ("Cue Exposure Therap*" OR "Cue exposure intervention*" OR "CET" OR "Exposure therap*" OR "Exposure intervention*" OR "Conditioning Therap*" OR "Conditioning intervention*" OR "Cue Exposure" OR "VR" OR "Virtual reality" OR "Desensitization, Psychologic" OR "Implosive Therapy" OR "Virtual Reality Exposure Therap*" OR "Virtual Reality Therap*" OR "Desensitization, Psychologic")) AND ("Craving" OR "Drive" OR "Motivation" OR "Alcohol drinking" OR "Alcohol consumption" OR "Alcohol intake" OR "Cocaine consumption" OR "Cocaine intake" OR "Cannabis consumption" OR "Cannabis intake" OR "Opioid consumption" OR "Opioid intake" OR "Amphetamine consumption" OR "Amphetamine intake" OR "Nicotine consumption" OR "Nicotine intake" OR "Ketamine consumption" OR "Ketamine intake" OR "Benzodiazepine consumption" OR "Benzodiazepine intake" OR "Benzodiazepine intake" OR "Drinking behavior" OR "Smoking" OR "Marijuana use" OR "drug-seeking behavior" OR "Motivation" OR "Drinking behavior" OR "Smoking" OR"Marijuana use" OR "drug-seeking behavior")

EMBASE

("Alcohol use disorder*" OR "AUD" OR "Alcohol Related Disorder*" OR "Alcohol-Related Disorder*" OR "Alcohol Induced Disorder*" OR "Alcohol-Induced Disorder*" OR "Alcohol-Dependen*" OR "Ethanol Dependen*" OR "Alcohol Abuse" OR "Ethanol Abuse" OR "Alcohol Addict*" OR "Alcohol Dependen*" OR "Alcohol Misuse" OR "Harmful Use of Alcohol" OR "Chronic Alcoholi*" OR "Alcoholic Intoxication" OR "Alcohol Intoxication" OR "Alcohol-Intoxication" OR "Binge Drink*" OR "Alcoholi*" OR "Problem Drinker" OR "Problematic Drinker" OR "Alcohol-Related Disorders" OR "Alcoholics" OR "SUD" OR "Drug abuse" OR "Drug addiction" OR "Cocaine" OR "Heroin" OR "Opioid*" OR "Nicotine" OR "cannabis" OR "tobacco" OR "TUD" OR "Addictive disorders" OR "addiction" OR "substance use disorders" OR "Substance Addiction" OR "Substance Dependence" OR "Drug Use" OR "Substance-Related Disorders" OR "Substance-Related Disorders") AND ("Cue Exposure Therap*" OR "Cue exposure intervention*" OR "CET" OR "Exposure therap*" OR "Exposure intervention*" OR "Conditioning Therap*" OR "Conditioning intervention*" OR "Cue Exposure" OR "VR" OR "Virtual reality" OR "Desensitization, Psychologic" OR "Implosive Therapy" OR "Virtual Reality Exposure Therap*" OR "Virtual Reality Therap*" OR "Desensitization, Psychologic")) AND ("Craving" OR "Drive" OR "Motivation" OR "Alcohol drinking" OR "Alcohol consumption" OR "Alcohol intake" OR "Cocaine consumption" OR "Cocaine intake" OR "Cannabis consumption" OR "Cannabis intake" OR "Opioid consumption" OR "Opioid intake" OR "Amphetamine consumption" OR "Amphetamine intake" OR "Nicotine consumption" OR "Nicotine intake" OR "Ketamine consumption" OR "Ketamine intake" OR "Benzodiazepine consumption" OR "Benzodiazepine intake" OR "Benzodiazepine intake" OR "Drinking behavior" OR "Smoking" OR "Marijuana use" OR "drug-seeking behavior" OR "Motivation" OR "Drinking behavior" OR "Smoking" OR"Marijuana use" OR "drug-seeking behavior")

Cochrane

"Alcohol use disorder*" OR "AUD" OR "Alcohol Related Disorder*" OR "Alcohol-Related Disorder*" OR "Alcohol Induced Disorder*" OR "Alcohol-Induced Disorder*" OR "Alcohol-Dependen*" OR "Ethanol Dependen*" OR "Alcohol Abuse" OR "Ethanol Abuse" OR "Alcohol Addict*" OR "Alcohol Dependen*" OR "Alcohol Misuse" OR "Harmful Use of Alcohol" OR "Chronic Alcoholi*" OR "Alcoholic Intoxication" OR "Alcohol Intoxication" OR "Alcohol-Intoxication" OR "Binge Drink*" OR "Alcoholi*" OR "Problem Drinker" OR "Problematic Drinker" OR "Alcohol-Related Disorders" OR "Alcoholics" OR "SUD" OR "Drug abuse" OR "Drug addiction" OR "Cocaine" OR "Heroin" OR "Opioid*" OR "Nicotine" OR "cannabis" OR "tobacco" OR "TUD" OR "Addictive disorders" OR "addiction" OR "substance use disorders" OR "Substance Addiction" OR "Substance Dependence" OR "Drug Use" OR "Substance-Related Disorders" OR "Substance-Related Disorders" OR “Substance-related disorders” AND "Cue Exposure Therap*" OR "Cue exposure intervention*" OR "CET" OR "Exposure therap*" OR "Exposure intervention*" OR "Conditioning Therap*" OR "Conditioning intervention*" OR "Cue Exposure" OR "VR" OR "Virtual reality" OR "Desensitization, Psychologic" OR "Implosive Therapy" OR "Virtual Reality Exposure Therap*" OR "Virtual Reality Therap*" OR "Desensitization, Psychologic" OR “Desensitization, Psychologic” AND "Craving" OR "Drive" OR "Motivation" OR "Alcohol drinking" OR "Alcohol consumption" OR "Alcohol intake" OR "Cocaine consumption" OR "Cocaine intake" OR "Cannabis consumption" OR "Cannabis intake" OR "Opioid consumption" OR "Opioid intake" OR "Amphetamine consumption" OR "Amphetamine intake" OR "Nicotine consumption" OR "Nicotine intake" OR "Ketamine consumption" OR "Ketamine intake" OR "Benzodiazepine consumption" OR "Benzodiazepine intake" OR "Benzodiazepine intake" OR "Drinking behavior" OR "Smoking" OR "Marijuana use" OR "drug-seeking behavior" OR "Motivation" OR "Drinking behavior" OR "Smoking" OR "Marijuana use" OR "drug-seeking behavior" OR “Motivation”

**Appendix 2** Sensitivity analyses excluding studies with <15 subjects in the intervention or control groups

Craving: The proportions of statistically significant findings and the direction of the findings when comparing cue exposure therapy (CET) and control interventions on craving outcomes across substance use disorders and delivery formats.

Consumption: The proportions of statistically significant findings and the direction of the findings when comparing cue exposure therapy (CET) and control interventions on consumption outcomes across substance use disorders and delivery formats.
