# Supplementary material for: The efficacy of conventional and technology assisted cue exposure therapy for treating substance use disorders: a qualitative systematic review
Source: Front Psychiatry. 2025 Mar 26;16:1544763. doi: 10.3389/fpsyt.2025.1544763 (PMC11979113; doi:10.3389/fpsyt.2025.1544763)
Supplement: Supplementary file 2 [file DataSheet2.docx]

| **Appendix 2**  **Supplementary Table 1.** Summary of all included studies | | | | | | | | | | | | | | | | | | |
| --- | --- | --- | --- | --- | --- | --- | --- | --- | --- | --- | --- | --- | --- | --- | --- | --- | --- | --- |
| **Substance** | **Author/**  **year** | **Study design** | **Age (mean), male (%)** | **SUD assess.** | **SUD pop. setting** | **Treatment as usual (TAU)** | | **Exp. group** | | **No. of sessions + duration** | **Cont. group** | | **Tx. goal** | **Outcome measures** | | **Follow-up** | **Main findings** | **Risk of bias (overall)** |
|  |  |  |  |  |  | **Psych.** | **Pharm.** | **n** | **Content** |  | **n** | **Content** |  | **Cravings** | **Consumption** |  |  |  |
| **NON-TECHNOLOGY CET** | | | | | | | | | | | | | | | | | | |
| Alcohol (n=13) | Rankin et al.  (1983) | RCT | Age NR, 100% male | NR | Clinical (I) | NR | None | 5 | In vivo (simple) + PRP | 6 sessions,  45 min/session,  within 8 days | 5 | Imaginal CET | RC | 10-point scale  (desire for a drink) | NA | Post-treatment | Craving was reduced in both groups (p<0.01). Exp. group reduced craving more than cont group, but p=NR. No effect size reported | High |
|  | McCusker et al.  (1995) | RCT | 35 years,  63% males | NR | Clinical (I) | AA + OPSET | None | 8 | In vivo (simple) + TAU | 5 sessions,  unknown duration, within 6 weeks | 8 | In vivo (simple, neutral cues) + TAU | RC | 5-point scale (craving/desire for a drink) | NA | Post-treatment | No difference in craving within or between groups. No effect size reported | Some concern |
|  | Sitharthan et al.  (1997) | RCT | Age NR,  79% males | NR | Clinical (O) | NR | None | 22 | In vivo (simple) + PRP | 6 sessions,  90 min/session, within 6 weeks | 20 | CBT | M | NR | PDQ: drinks per months and drinks per drinking day (DDD) | 6 mo. | Consumption was reduced for both groups (all p-values<0.001). Consumption was more reduced after exp.- vs. cont. group (all p-values<0.05). No effect size reported. | Some concern |
|  | Staiger et al.  (1999) | RCT | 35 years, 100% males | CD: DSM-III | Clinical (I) | OPSET | None | 25 | In vivo (simple) + TAU | 5 sessions, 1 hour/session, within 5 days | 25 | In vivo (simple, neutral cues) + TAU | RC | 5-point Likert scale  (urge to drink) | NA | Post-treatment | Craving was reduced in exp. group (p=0.01). Craving was more reduced after exp.- vs. cont. group (p<0.004). No effect size reported. | Some concern |
|  | Heather et al. (2000) | RCT | 41 years,  75% males | NR | Clinical (I) | NR | None | 48 | In vivo (simple) +PRP | 16 sessions, 90 min/session, within 16 weeks | 43 | CBT | M | NR | Form 60: drinks per drinking day (DDD), percentage days abstinence PDA) | Post treatment., and 6 mo. | Consumption was reduced for both groups (p=0.0001), with no difference between groups. No effect size reported. | Some concern |
|  | Rohsenow et al. (2001) | RCT | Age NR,  78% males | CD: DSM-III | Clinical (I) | AA + OPSET | NR | 100 (exp.+ cont.) | In vivo (simple) + Imaginal CET + USCS + TAU | 10 sessions,  50 min/session, within 8 weeks | NR | MRT + TAU | M, RC | 11-point Likert scale (urge to drink) | TLFB: abstinence, percentage of heavy drinking days (HDD), drinks per day (DD) | Post-treatment (craving), 3-, 6-, and 12 mo. (consumption) | Craving was reduced in both groups (p<0.001), with no difference between groups.   PHDD was reduced for both groups at 6.- and 12. mo. FU, (p<0.05), and DDD at 12-month FU. Exp. group reduced DDD and PHDD: among non-abstainers, more than cont. group at 12-month FU (p<0.05) No effect size reported. | Some concern |
|  | Dawe et al.  (2002) | RCT | 41.8 years, 61% males | NR | Clinical (O) | NR | NR | 50 | In vivo (simple) + PRP | 8 sessions | 50 | CBT | M | NR | Form 90: drinks per day (DD), drinks per drinking days (DDD), heavy drinking days (HDD) | 8 mo. | Consumption was reduced for both groups (p<0.1), with no difference between groups. No effect size reported. | Some concern |
|  | Kavanagh et al. (2006) | RCT | 43.2 years, 44% males | CIDI: DSM-IV | Clinical (O) | CBT | NR | 108 | G1: In vivo (complex) + Imaginal CET + PRP + TAU G2: In vivo (complex) + Imaginal CET + PRP + recall unpleasant experience + TAU | 8 sessions,  75 min/session, within 10 weeks | 55 | TAU | M, RC | 101-point scale  (want to drink) | PD: drinks per day (DD) | Post-treatment (craving and consumption), 3, 6, 9, and 12 mo. (consumption) | Craving was reduced in both exp. groups (all p-values <0.001), with no difference between the two exp. groups.  Consumption was reduced in all groups at all assessments (all p-values<0.001). Consumption was more reduced after cont.- vs. exp. groups (p<0.001). No effect size reported. | Some concern |
|  | Vollstädt-Klein et al. (2011) | RCT | 46.5 years, 63% males | CD: DSM-IV | Clinical (O) | OPSET | None | 15 | In vivo (simple) + Imaginal CET + TAU | 9 sessions,  60-90 min/session within 3 weeks | 15 | TAU | RC | VAS: 101-point | NA | Post-treatment | Craving was reduced in both groups (p=0.005), but no difference between groups. No effect size reported. | Some concern |
|  | Monti et al.  (1993) | CT | 40.2 years, 100% male | CD: DSM-III | Clinical (I) | OPSET | None | 22 | In vivo (simple) + Imaginal CET + USCS + TAU | 6 sessions,  55 min/sesion, within 2 weeks | 18 | TAU | A, RC | 10-point Likert scale (urge to drink) | TLFB: percentage days abstinent (PDA), drinks per drinking day (DDD) | Post-treatment (craving), 3-, and 6 mo. (consumption) | Craving was reduced in both groups (all p-values<0.003). Craving was more reduced in exp. vs. cont. group (p<0.001)  Consumption was not tested within groups, but exp. group reduced PDA more than con. group at 6-mo. FU (p<0.05). No effect size reported. | Some concern |
|  | Drummond et al.  (1994) | CT | 44 years, 100% males | CD: DSM-III | Clinical (I) | AA + OPSET | Yes: NS | 20 | In vivo (simple) + TAU | 10 sessions,  40 min/session, within 10 days | 15 | Relaxation + TAU | A | NR | SART: categorical drinking levels (ranging from 0 = no drinking to 5= > 40 units), relapse to heavy drinking and total number of drinks score. | Post-treatment (craving), 1-, 3-, and 6 mo. (consumption) | No within group analyses was reported. Exp. group more days before relapse to heavy drinking (p<0.01) and decreased total number of drinks (p<0.01) more than cont. group at 6-month FU. No effect size reported. | High |
|  | Monti et al.  (2001) | CT | 39.2 years, 76% males | SCID: DSM-IV | Clinical (I/O) | AA + OPSET | Yes (except disulfiram). After discharge, subjects were randomized to 12 w. of naltrexon or placebo. | 77 | In vivo (simple) + Imaginal CET + USCS + CST + TAU | 8 sessions,  1 hour/session, within 8 weeks | 88 | MRT + TAU | A | NR | TLFB: drinking days (DD), drinks per drinking day (DDD), percentage relapsed to heavy drinking ((HD), heavy drinking days (HDD) | Post-treatment, 3-, 6-,  and 12 mo. | No craving outcomes reported and no within group analyses on neither craving nor alcohol consumption. Exp. group. reduced percentage relapsed to HDD and percentage of HDD more than cont. group at 6- and 12 mo. FU (all p-values<0.05). Small effect size at 6 mo. FU: 0.17. Small effect size at 12 mo. FU: 0.19. | High |
|  | Loeber et al.  (2006) | CT | 46.3 years, 57% males | CD: DSM-IV | Clinical (I) | OPSET | None | 31 | In vivo (simple) + Imaginal CET + USCS + TAU | 9 session,  90 min/session,  within 3 weeks | 32 | CBT | A, RC | ACQ | TLFB: time to relapse to heavy drinking (HD), cumulative abstinence, total number of drinks | Post-treatment (craving), 1-, 2-, 3-, 4-, 5-, and 6 mo. (consumption) | Craving was reduced in both groups (all p-values<0.05). There was no difference between groups.   Abstinence increased and total number of drinks decreased up to 6 mo. FU (all p-values<0.001), with no difference between groups. No effect size reported. | High |
| Nicotine  (n=5) | Corty et al.  (1984) | RCT | 36 years,  44% male | NR | Sub-clinical (O) | None | None | 21 | In vivo (simple) + Imaginal CET | 8 sessions | 18 | Rapid smoking | M | NR | PD: cigarettes per day (CD) | Post-treatment, 1-, 3-, and 6 mo. | Consumption was reduced in both groups at post-treatment and 6 mo. FU. No p-values reported. No effect size reported. | High |
|  | Brandon et al.  (1987) | RCT | 31.30 years, gender: NR | NR | Sub-clinical (NR) | CBT + BT | None | 18/18 | G1: In vivo/vitro: NS + USCS + TAU G2: In vivo/vitro: NS + USCS + rapid smoking + TAU | 4 sessions,  1.25 hours/session, within 10 weeks | 19 | TAU | A | NR | N-SART: abstinence (verified by collaterals) | Post-treatment, 1-, 2-, 3-, 4-, and 12 mo. | No within group analysis reported. Rate of abstainers increased more in exp. vs. cont. group at 3 mo. (p<0.05). Pre-session smoking reduced more in exp. vs. cont. group at 4 mo. (p<0.05). No difference between exp. groups G1 and G2. No effect size reported. | Some concern |
|  | Niaura et al.  (1999) | RCT | 43.5 years, 50% male | NR | Sub-clinical (NR) | OPSET | NR | 31/31 | G1: In vivo (simple) + Imaginal CET + CBT + TAU G2: In vivo (simple) + Imaginal CET + CBT + nicotine gum + TAU | 5 sessions, 1.25 hours/session, within 4 weeks | 32/35 | G3: Brief CBT + TAU G4: CBT + nicotine gum + TAU | A, RC | 11-point-scale (urge to smoke) | SART: abstinence and time to relapse (24-hour abstinence verified by exhaled CO) | Post-treatment (craving, consumption), 1-, 3-, 6-, and 12 mo. (consumption) | Craving and consumption (abstinence and time to relapse) was reduced in all groups (all p-values<0.01), but no differenece between groups. No effect size reported. | Some concern |
|  | Morganstern et al.  (1969) | CT | Age and gender: NR | NR | Sub-clinical (U/L) | None | None | 8 | In vivo (simple) + Imaginal CET + deep muscle relaxation | 6 sessions,  50 min/session, within 5 weeks | 32 | No treatment | A | NA | SART: abstinence, cigarettes per day (CD) | Post-treatment | 3/8 were abstinent and all reduced daily consumption in exp. group. 1/32 reduced daily consumption in cont. group. No p-values reported. No effect size reported. | High |
|  | Raw et al.  (1980) | CT | 39.5 years, 40% male | NR | Sub-clinical (O) | None | None | 17 | G1: In vivo (simple) | 7 sessions, 45 min/session, within 4 weeks | 32 | Rapid smoking + MI + USCS | M, RC | 7-point scale (desire for a smoke) | PD: cigarettes per day (CD) | Post-treatment, 3-, 6-, and 12 mo. | No difference in craving within or between groups.   Consumption was reduced for all groups (all p-values<0.001), but no differences between groups. No effect size reported. | High |
| Opioids  (n=2) | Dawe et al.  (1993) | RCT | 29 years, gender NR | NR | Clinical (I) | CBT | None | 18 | In vivo (complex) + Imaginal CET + TAU | G1: 6 sessions, 41-80 min/session, within 12 weeks G3: 6 sessions, 41-80 min/session, within 3 weeks | 25 | TAU | A, RC | 9-point scale (craving for opiates) | SART: abstinence, daily use, days of use in last month, total substance use, number of days to first use/relapse | Post-treatment (craving), 6-w., and 6-mo. (consumption). | Craving was reduced in all groups: all p-values<0.0001, with no difference between groups.  No within group analyses of consumption. No difference between groups on any consumption outcome. No effect size reported. | High |
|  | Marissen et al.  (2007) | RCT | 33.6 years, 89% male | CD: DSM-IV | Clinical (I) | None | None | 65 | In vivo (simple) + USCS | 9 sessions, 1 hour/ sessions, within 3 weeks | 62 | OPSET | A, RC | VAS: 101-points (current craving), DDQ (Desire to use heroin) | SART: relapse | Post-treatment (craving) and 3 mo. (craving and consumption). | Craving was reduced in both groups, but no difference between groups (p-values:NR)  No within group analyses of consumption. Relapse was lower in cont. vs. expt. (p=0.01). No effect size reported. | High |
| Mix of SUDs  (n=1) | Havermans et al.  (2007) | RCT | 37 years,  81% male | CD: DSM-IV (AUD n=36; CSUD: cocaine: n=13; OUD: NS: n=11; CUD cannabis:n=4; ASUD: benzodiazepines n=2) | Clinical (I) | CBT | No anti-craving medication | 36 | In vivo (simple) + Imaginal CET + TAU | 11 session,  1 hr/session,  within 4-5 weeks | 34 | Relaxation training + TAU | RC | VAS: 101-point-scale  (urge to drink) | NA | Post-treatment | Craving was reduced in both groups (p-value=0.003), but no difference between groups. No effect size reported. | Some concerns |
| **TECHNOLOGY-ASSISTED CET** | | | | | | | | | | | | | | | | | | |
| Alcohol  (n=9) | Geisel et al.  (2016) | RCT | 43.1 years, 73.2% male | CD: ICD-10 | Clinical (O) | NR | Yes (usage of antidepressant,  naltrexon, benzodiazeoines and others was reported) | 30 | In vivo (simple, pictures on monitor) + TAU | 6 session,  55 min/sesion, within 2 weeks | 14 | In vivo (simple, neutral cues) + TAU | RC | AUQ (want to drink),  VASC: 101-point (desire to drink) | NA | Post-treatment | Craving was reduced in a sub-group of the cont. group (p⋜0.024), with more reduction in a sub-cont.-group vs. a sub-exp.-group (AUQ: p=0.005; VASQ: p=0.004). No effect size reported. | Low |
|  | Mellentin et al. (2019) | RCT | 46.3 years, 42.3% male | CD: ICD-10 | Clinical (O) | CBT | Yes  (e.g., disulfiram, acamprosate, neltrexone) | 54/54 | Aftercare interventions: 1: In vivo (simple) + Imaginal CET + USCS + TAU 2: In vivo on app (simple) + Imaginal CET + USCS on a mobile app. + TAU | 4 sessions,  2 hrs/session within 8 weeks vs. max 32 sessions, 15 min/session, within 8 weeks | 56 | Aftercare as usual + TAU | M | VAS: 11-point (craving to drink) | TLFB: sensible drinking,  abstinence, drinking days (DD), heavy drinking days (HDD) | Postaftercare and 6 mo. | Craving pattern did not differ significantly between groups.increased for all groups Consumption increased for all groups (all p-values⋜0.006), with no difference between groups. No effect size reported. | Low |
|  | Hernandes-Serrano et al.  (2020) | RCT | 54.6 years,  50% male | CD: DSM-V | Clinical (O) | CBT + MI | None | 35 | In vivo (complex, VR) + TAU | 6 sessions,  50 min/session,  within 6 weeks | 44 | TAU | RC | MACS-VR: 41-point, desire to drink during VR and behavioral disinhibition | NA | Post-treatment, and 3 days after the final treatment (post-test) | Craving was only reduced in the exp. group at post-treatment (p = 0.003), and the exp. group reduced cravings more than cont. group (p = 0.001). No effect size reported. | High |
|  | Zhang et al. (2023) | RCT | 35.1 years, 100% male | CD: ICD-10 | Clincal (I) | NR | Benzodiazepines, vitamine B and C | 29 | In vivo (complex, VR) + TAU | 8 sessions, 8 min/session, withing 3 weeks | 28 | TAU | RC | VAS: 11-point scale (craving to drink) | NA | Post-treatment | Craving was significantly reduced in the exp. group (p<0.05).  Craving was significantly more reduced in the exp. group vs. the cont. group (p<0.05). No effect size reported. | High |
|  | Weber et al. (2023) | RCT | 44.04 years, 65% male | CD: DSM-V | Clinical (I/O) | NR | Alcohol detoxification | 31 | In vivo (complex, computer) +TAU | 6 sessions, 30 min/session, within 2 weeks | 31 | TAU | RC | OCDS: 56-point scale (Strength and frequency of craving) | NA | Post-treatment | Craving was significantly reduced in both groups (p=0.04) with no difference between groups. No effect size reported. | High |
|  | Thaysen-Petersen et al. (2024) | RCT | 46 years, 56% male | CD: ICD-10 | Clinical (O) | CBT | None | 5 | In vivo (VR, complex) +TAU | 3 sessions, 45-60 min/session, within 3 weeks | 5 | TAU | RC, M | PACS (craving during the last week) | TLFB: Units/month, heavy drinking days (HDD) | Post-treatment and 1 mo. | Craving and consumption were reduced in both groups. No p-values reported and no between group analysis. No effect size reported. | High |
|  | Ng et al. (2024) | RCT | 36.77 years, 100% male | CD: DSM-V | Clinical (I/O) | OPSET | Yes (ong-term medication to deal with craving) | 31 | In vivo (complex, laptop/projector) + phone calls + text message reminders + TAU | 8 sessions, 45-60 min/session, within 2 weeks | 30 | TAU | A, RC | PACS (craving during the last week) | 90-AQ: abstinence, drinking days per month, quantity of drinking per month | 3 mo. | No p-values on within group data.  Abstinence was increased and consumption (quantity and frequency) and craving was significantly more reduced in exp. vs. con. group (p<0.0001). No effect size reported. | High |
|  | Lee et al.  (2009) | CT | 38.5 years,  100% males | CD: DSM-IV | Clinical (I) | NR | None | 20 | In vivo (complex, VR/projector) + aversion | 10 sessions,  1 hour/session within 5 weeks | 18 | CBT | RC | VAS: 11-point (craving to drink) | NA | Post-treatment | Craving was reduced in both groups (all p-values<0.01), and the exp. group reduced craving more than cont. group (p=0.01). No effect size reported. | High |
|  | Nattala et al.  (2018) | CT | 37.9 years,  100% male | CD: ICD-10 | Clinical (I) | CBT + BT + OPSET | Yes  (long-term medication to deal with withdrawal and craving) | 45 | In vivo (complex, projector and laptop) + USCS | 8 sessions,  45-60 min/session, within 3 weeks | 45 | TAU | M | NR | Form 90: drinking days (DD), time to first drink-, total number of drinks | 1, 2, 3, 4, 5 and 6 mo. | Consumption (quantity and number of drinking days) was significantly reduced in exp. group (P-value: NR). No data on cont. group. Consumption was lower in exp.- vs. cont. group at 6 mo. FU (all p-values⋜0.002). No effect size reported. | High |
| Nicotine  (n=9) | Girard et al.  (2009) | RCT | 44 years, 43% male | NR (FTND≥4) | Sub-clinical (O) | OPSET | No medication affecting the central nervuos system | 46 | In vivo (simple, VR) + crushing cigarettes + TAU | 12 sessions, 30 min/session, within 12 weeks | 45 | In vivo (complex, neutral, collecting balls in VR) + TAU | M | NR | Daily smoker’s journal: abstinence and cigarettes per day (CD) (verified by expired CO) | Post-VR, Post-treatment (consumption, FTND), and 6 mo. (consumption) | No within group p-values reported. Abstinence was increased and number of cigarettes was reduced more in exp. group vs. cont. group at post-treatment and 6 mo. (all p<0.05). No effect size reported. | High |
|  | Bordnick et al.  (2012) | RCT | 47 years, 52% male | SCID: DSM-IV | Clinical (O) | None | Nicotine Replacement Therapy (NRT); No psychotropic medication | 21 | In vivo (complex, VR) + CBT + TAU | 10 sessions, 1 hour/sessions, within 10 weeks | 25 | TAU | M, RC | QSU-Brief | TLFB: cigarettes per day, total number of cigarettes | Post-treatment. (craving, consumption), 1-, 2-, 3-, and 6 mo. (consumption) | No within group p-values reported.   Craving (QSU-Brief) was reduced more in exp. group vs. cont. group at Post-treatment (p=0.012).  Consumption was reduced more in exp. group vs. cont. group at post-treatment. (p<0.05), 2 mo.- (p <0.05) and 6 mo. FU (p<0.01). No effect size reported. | Some concern |
|  | Culbertson et al.  (2012) | RCT | 42.1 years, 91% male | CD: DSM-IV | Clinical (NR) | CBT | NR | 5 | In vivo (complex, VR) + TAU | 16 sessions,  30 min/session, within 8 weeks | 6 | In vivo (complex, neutral cues, VR) + TAU | M, RC | UTS | SART: abstinence, cigarettes per day (CD) | Post-treatment | Craving was reduced in both groups (all p-values<0.05), but no difference between groups.  No within group data on consumption. Consumption was more reduced in exp. group vs. cont. group (p=0.034). Rate of abstainers was increased in exp. group vs. cont. group (p<0.015). No effect size reported. | Some concern |
|  | Malbos et al.  (2018) | RCT | 49 years, 28% male | CD: DSM-V | Clinical (O) | CBT | NR | 61 (exp.+ cont.) | In vivo (complex, VR) + TAU | 8 sessions, 45 min/session, within 8 weeks | NR | Imaginal CET + TAU | RC | TCQ, VAS: 100-point-scale (level of craving) | NA | Post-treatment | Craving (TCQ and VAS) were reduced in both groups (all p-values<0.001), but no difference between groups. No effect size reported. | High |
|  | Pericot-Valverde et al.  (2019) | RCT | 39.4 years, 36% male | SCID: DSM-IV | Clinical (O) | CBT | No other smoking cessation tx. | 50 | In vivo (complex, VR) + TAU | 6 sessions, 90 min/session, within 6 weeks | 52 | TAU | A, RC | 101-point-scale (only measured in exp. group) | SART: abstinence, cummulative abstinence, relapse rate | Post-treatment (abstinence), 1-, 6-, and 12 mo. FU (abstinence, continuous abstinence, relapse rate) | No within group results reported. Craving was more reduced in exp. group vs. con. group (p<0.001.)  Relapse rate lower in cont.. group vs. exp.. group at 12 mo. FU (p=0.029). No effect size reported. | Some concern |
|  | Goldenhersch et al.  (2020) | RCT | 43.25 years, 52% male | NR (min. 5 cigarets/day) | Sub-clinical (NR) | None | NR | 60 | In vivo (complex, VR (mobile phone + cardboard headset)) + mindfulness + peer-to-peer support (app group chat) | 21 sessions, approx. 20 min/session, within 3 weeks | 60 | Self-help manual | M, RC | QSU | SART: abstinence, cummulative abstinence and cigarettes per day (CD) | Post-treatment (craving, consumption), and 3 mo. (consumption) | Craving was reduced at post-treatment in the exp. group (p-values<0.005). No within group analyses were reported for the cont. group or between group analysis.  Consumption (all measures) was reduced at post-treatment in exp. group (all p-values p<0.001). No within group analyses were reported for the cont. group. Abstinence rate was higher in exp. vs. cont. group at post-treatment (p=0.004). No effect size reported. | Some concern |
|  | Pollak et al.  (2021) | RCT | 43.2 years, 25% male | NR (light smokers: 1-10 cigarets/day) | Sub-clinical (NR) | CBT | NR | 13 | In vivo (complex, computer) + TAU | 5 sessions, within 5 weeks | 11 | TAU | A | NR | N-SART: abstinence | Post-treatment | The rate of abstainers was increased in both groups: exp. Group: 4/13; cont. group: 3/11. No p-values reported. No effect size reported. | High |
|  | Malbos et al. (2022) | RCT | 47.65 years, 29% male | DSM-V | Clinical (O) | CBT | NR | 50 | In vivo (complex, VR) + TAU | 8 sessions, 45 min/session, within 8 weeks | 50 | TAU | A, RC | FTCQ-12: 73-point-scale, VAS: 100-point-scale | SART: relapse (verified by expired CO) | Post-treatment | Craving (all measures) decreased in both groups (p<0.001). Relapse was lower in exp. group compared to cont. (p =0.026). No effect size reported. | High |
|  | Park et al.  (2014) | CT | 31.9 years, 100% male | NR (FTND) | Sub-clinical (O) | None | None | 15 | In vivo (complex, VR) | 4 sessions, 25 min/session, within 4 weeks | 15 | CBT | M, RC | QSU | PD: cigarettes per day (CD) | Post-treatmentand 2 mo. | Craving was not reduced neither within nor between-group. Consumption was reduced in both groups (all p-values<0.05), but no difference between groups. No effect size reported. | High |
| COCAINE AND OTHER  STIMULANT USE DISORDERS  (n=3) | O'Brien et al.  (1990) | RCT | Age range: 28-53 years, 100 % male | NR (cocaine) | Clinical (I/O) | OPSET | NR | 30 in total (exp.- + cont. group) | In vivo (complex, videotape) + TAU | 15 sessions,  1 hr/sessions, within 2 weeks | NR | TAU | RC | 10-point scale  (degree of craving) | NA | Post-treatment | Craving was reduced in both exp. Groups (all p-values<0.0001). No results from con. groups or comparison between groups. No effect size reported. | High |
|  | Wang et al.  (2019) | RCT | 33.8 years, 100% male | CD: DSM-V (Methamphetamine) | Clinical (O) | OPSET | None | 31 | In vivo (complex, VR) + aversion + TAU | 6 sessions, within 3 weeks | 30 | TAU | RC | VAS: 11-point-scale (craving for meth) | NA | Post-treatment | Craving was reduced in exp. group (p-value<0.001). Craving in exp. group was lower than con. group (p<0.001). | High |
|  | Ji et al. (2023) | RCT | 23 years, 100% female | CD: ICD-10 (Methamphetamine) | Clinical (I) | NR | Detoxification management | 30 | In vivo (complex, VR) + MI + TAU | 8 sessions, 60 min/session, within 4 weeks | 30 | TAU | RC | VAS: 101-point scale (degree of craving) | NA | Post-treatmentand 1 mo, | Craving was reduced in exp. group at both FU visits (p<0.01).  Craving was more reduced in exp. group vs. cont. group (p<0.01). No effect size reported. | Some concern |
| Opioids  (n=2) | Aragón et al.  (2005) | RCT | 27.4, 70% male | NR | Clinical (O) | CBT | NR | 12 | In vivo (complex, video) + TAU | 12 sessions, 60-75 min/session,  within 4 weeks | 12 | TAU | RC | 101-point scale (degree of craving) | NA | Post-treatment and 6 mo. | Craving was not reduced in both groups. Groups were not compared. No effect size reported. | High |
|  | Du et al.  (2014) | RCT | 34.6, 47% male | SCID: DSM-IV | Clinical (I) | OPSET | NR | 23 | In vivo (complex, video) + Imaginal CET + biofeedback therapy + TAU | 12 sessions, 60 min/ sessions within 8 weeks | 22 | TAU | RC | VAS: 101-point  (urge to use heroin) | NA | Post-treatment | Craving decreased in exp. group at post-treatment (p<0.01). No change was found i cont. group. Craving was more reduced in the exp. group vs. cont. group at post-treatment (all p-values<0.01). No effect size reported. | High |
| ABBREVIATIONS: A: Abstinence; ACQ: Alcohol Craving Questionnaire; ADS: Alcohol Dependence Scale; ASI: Addiction Severity Index; AUD: alcohol use disorder; AUDIT: AQ: Alcohol Questionnaire; AUQ: Alcohol Urge Questionnaire; ASUD: BSCT: Behavioral Self-Control Training; BT: Behavioral therapy; CBT: cognitive behavior therapy; CCQ-Now: Cocaine Craving Questionnaire-Now; CIDI: Composite International Diagnostic Interview; CM: Contingency Management; CRS: Craving Reactivity Scale; CST: Communication Skill Training; DDQ: Daily Drinking Questionnaire; DSM-IV: The Diagnosis and Statistical Manual of Mental Disorders, 4th Edition; ED: Education discussion; FTCQ-12: French Tobacco Craving Questionnaire; FTND: Fagerstrom Test of Nicotine Dependence; I: In-patient; M: Moderation; MACS-VR: Multidimensional Alcohol Craving Scale Virtual Reality (modified from MACS); mo.: months; MRT: Meditation Relaxation Techniques; NRT: Nicotine Replacement Therapy; O: OCDS: Obsessive Compulsive Drinking Scale; Out-patient; OPS: Other psychological treatment; PACS: Pens Alcohol Craving Scale; PDQ: Problem Drinking Questionnaire; QSU: Questionnaire of Smoking Urge; RC: Reduce craving; SADQ: Severity of Alcohol Dependence Questionnaire; STNR: Standard Treatment Not Specified; SUD: Substance Use Disorders; TCQ: Tobacco Craving Questionnaire; TLFB: Time-Line Follow-Back Interview; U/L: University/Laboratory; USCS: Urge Specific Coping Skill; UTS: Urge To Smoke; W: Weeks | | | | | | | | | | | | | | | | | | |
